# Comparative Tissue Identification and Characterization of Long Non-Coding RNAs in the Globally Distributed Blue Shark *Prionace glauca*

**DOI:** 10.3390/life14091144

**Published:** 2024-09-11

**Authors:** Scarleth Bravo, Patricia Zarate, Ilia Cari, Ljubitza Clavijo, Ignacio Lopez, Nicole M. Phillips, Rodrigo Vidal

**Affiliations:** 1 Laboratory of Genomics, Molecular Ecology and Evolutionary Studies, Department of Biology, Universidad de Santiago de Chile, Santiago 9160000, Chile; scarleth.bravo@usach.cl (S.B.); ignacio.lopez.r@usach.cl (I.L.); 2 Departamento de Oceanografía y Medio Ambiente, División de Investigación Pesquera, Instituto de Fomento Pesquero, Valparaíso 2361827, Chile; patricia.zarate@ifop.cl (P.Z.); ilia.cari@ifop.cl (I.C.); ljubitza.clavijo@ifop.cl (L.C.); 3 School of Biological, Environmental, and Earth Sciences, University of Southern Mississippi, Hattiesburg, MS 39406, USA; n.phillips@usm.edu

**Keywords:** elasmobranch, transcriptome, ncRNA, lncRNA, differential expression

## Abstract

Long non-coding RNAs (lncRNAs) are involved in numerous biological processes and serve crucial regulatory functions in both animals and plants. Nevertheless, there is limited understanding of lncRNAs and their patterns of expression and roles in sharks. In the current study, we systematically identified and characterized lncRNAs in the blue shark (*Prionace glauca*) from four tissues (liver, spleen, muscle, and kidney) using high-throughput sequencing and bioinformatics tools. A total of 21,932 high-confidence lncRNAs were identified, with 8984 and 3067 stably and tissue-specific expressed lncRNAs, respectively. In addition, a total of 45,007 differentially expressed (DE) lncRNAs were obtained among tissues, with kidney versus muscle having the largest numbers across tissues. DE lncRNAs trans target protein-coding genes were predicted, and functional gene ontology enrichment of these genes showed GO terms such as muscle system processes, cellular/metabolic processes, and stress and immune responses, all of which correspond with the specific biological functions of each tissue analyzed. These results advance our knowledge of lncRNAs in sharks and present novel data on tissue-specific lncRNAs, providing key information to support future functional shark investigations.

## 1. Introduction

Sharks (Superorder selachii) are an ancient group of cartilaginous fish with origins dating back to the Silurian–Devonian periods (~420 million years before present, MYBP), with modern forms arising during the Jurassic period (195 MYBP) [1]. During this extensive evolutionary time, sharks survived at least five mass extinction events [2], which in some cases were followed by a rapid diversification of surviving lineages [3]. For this reason, sharks may be considered a resilient group with potentially significant genomic characteristics that have allowed them to overcome challenges throughout their evolutionary history, such as adaptation to a changing environment and/or resistance to disease.

Following the most recent mass extinction at the end of the Cretaceous, and still today, the most diverse shark order is the *Carcharhiniformes*, comprising 337 of the 536 described extant species. The blue shark (*Prionace glauca*) (Linnaeus 1758) is a wide-ranging pelagic carcharhinid found at tropical and temperate latitudes in all oceans [4]. This species was assessed globally as Near Threatened on the International Union for Conservation of Nature (IUCN) Red List of Threatened Species due to population declines resulting from mortalities in fisheries [5]. Blue sharks are harvested for fins and meat via direct fisheries and, when caught, as bycatch in both commercial and small-scale fisheries [6,7]. As apex predators, blue sharks are critical to healthy ecosystem function and structure through increasing biodiversity and buffering against invasive species, transmission of disease, and climate change [8]. Additionally, as upper tropic level predators, blue sharks can be considered sentinel species of marine pollution as they are subject to bioaccumulation and biomagnification of contaminants in their tissues, including heavy metals and chemicals [9].

One early response of marine organisms to adapt to environmental changes and stress is alterations in the dynamics of transcript levels of particular genes [10]. It is widely accepted that RNA-sequencing (RNA-seq) is the principal sequencing technology used to understand the composition of transcriptomes and gene expression profiles in non-model species, such as blue sharks. Various studies have reported novel information on the transcriptomes of sharks [11,12,13,14]; however, these studies have focused primarily on the coding parts of the transcriptome. Several studies on the complexity of eukaryotic transcriptomes have revealed that a sizeable portion of the genome gives birth to non-coding RNA (ncRNA) genes [15]. A class of ncRNA that has received considerable attention in recent years is the long non-coding RNA (lncRNA). LncRNAs correspond to DNA sequences that encode transcripts larger than 200 nucleotides with low to null protein-coding potential. They may interact with molecules such as DNA, RNA, and proteins, regulating crucial molecular processes such as chromatin modeling, transcription, post-transcriptional regulation, translation, and post-translational modification [16]. Several studies have reported that lncRNAs are broadly distributed in diverse regions of vertebrate genomes, including retrotransposons in protein-coding genes, pseudogenes, introns, natural antisense transcripts, and intergenic regions [17]. Accumulating evidence shows that lncRNAs perform a significant role as gene expression regulators during stages of development of organs and in response to environmental changes, among others [18]. In this context, the regulatory effectiveness of lncRNAs is linked to its expression. Many lncRNAs display a tissue-specific profile of expression, generating meaningful clues about their specific activities within tissues. However, there are also stably expressed lncRNAs that are fundamental to the preservation of elementary cellular activities, regardless of tissue origin [19].

Due to the regulatory role of lncRNAs in several biological processes of metazoans, there are numerous studies devoted to the identification and characterization of lncRNAs in animals, plants, and teleost fishes (e.g., [20,21]), but such works in sharks are scarce [22,23]. Although lncRNAs and coding-protein genes share several molecular characteristics [24], they exhibit different sequence attributes that have been exploited to build new statistical deep learning models to evaluate the coding potential of any given transcript in organisms lacking reference genomes, obviating the constraints of database-based annotation approaches [25]. Here, we identify and characterize tissue-specific (TS) and stably expressed lncRNAs across four tissues of the blue shark using high-throughput RNA sequencing technology. Present results provide valuable information for understanding the regulation and function of lncRNAs in sharks.

## 2. Materials and Methods

### 2.1. Tissue Sampling and RNA Isolation

Tissue samples from blue sharks were obtained opportunistically by on-board fishery observers from IFOP (Instituto Fomento Pesquero, Valparaiso, Chile) during Chilean pelagic longline fisheries in the eastern Pacific Ocean in July 2022 (Exempt Resolution No. E-2022-071). The individuals sampled correspond to organisms that died during the fisheries process. Tissue samples from the kidney, spleen, liver, and muscle from each of four juvenile blue sharks (two males and two females; mean size of 98.6 cm fork length ±34.3 SD) were collected and immediately preserved in Allprotect Tissue Reagent (Qiagen, Qiagen, Germantown, MD, USA). Total RNA was isolated from each tissue using Trizol (Invitrogen, Carlsbad, CA, USA) and RNeasy Mini KIT (Qiagen, Germantown, MD, USA), and genomic DNA residue was removed with TURBO DNAse treatment (Invitrogen, Invitrogen, Carlsbad, CA, USA) according to the manufacturer’s instructions. The qualities, concentrations, and integrities of RNA extracts were evaluated with a Qubit 3.0 Fluorometer (Life Technologies, Carlsbad, CA, USA; Qubit RNA HS kit) and Bioanalyzer 2100 (Agilent Technologies, Santa Clara, CA, USA; RNA Nano 6000 Assay Kit).

### 2.2. Sequencing and lncRNA Identification

A total of 1.7 µg RNA per sample was used for rRNA elimination employing the Ribo-Zero rRNA Removal Kit (Epicentre, Madison, WI, USA). The NEBNext Ultra™ II Directional RNA Library Kit for Illumina (New England Biolabs, Ipswich, MA, USA) was used for the construction of 16 libraries (i.e., four tissues from each of four individuals), which were sequenced on an Illumina HiSeq 2500 sequencer (pair end-150 bp). The qualities of the raw reads of each library were checked using FastQC v-0.12.0 (Andrews, 2010) software. High-quality clean reads were collected with Trimmomatic v-0.38 software (Bolger et al., 2014) by eliminating reads with adapters (or poly-*N*) and reads that were low-quality or too short (<97 bp). To remove potential exogenous contamination in the dataset, Kraken 2 [26] was used with a custom database built with all sequences of plasmids, viruses, fungi, bacteria, protozoa, and archaea in RefSeq (release 212). The clean reads of the different tissues were combined and assembled, without genome guidance, with Trinity v-2.8.4 separately (no_normalize_reads- and the rest of parameter default) [27]. The assembled transcripts for the four tissues were clustered into superTranscripts (Trinity “gene”), obtaining the final assembled blue shark transcriptome. To evaluate the assembly quality, we used BUSCO v-5 (Benchmarking Universal Single-Copy Orthologs) (eukaryota_odb10 lineage) [28] and rnaQUAST v-2.2.3 [29]. To identify high-confidence blue shark lncRNAs, we applied several filters. First, we used Transdecoder v-5.7.0 (default parameters) (https://github.com/TransDecoder/TransDecoder, accessed on 14 March 2023) to search for open reading frames (ORFs). Second, all superTranscripts and ORFs were searched against NCBI/non-redundant and UniProt/UniRef90 protein databases (threshold = 1 × 10^−6^), and sequences matching with proteins were eliminated. Third, all potential non-coding RNAs were searched against the RFAM database v-14.9 [30] by Infernal v-1.1.4 (default parameters) [31], and any significant match reported was removed. To assess the coding potential of the filtered dataset, two novel deep learning packages, LncADeep v-1.0 [32] and RNAsamba [33], were used. Deep learning models outperform widely used approaches to identify lncRNAs, such as logistic regression (e.g., CPAT) and support vector machines (e.g., CPC2) [25]. If a superTranscript was assigned as coding by at least one of these two packages, it was treated as a possible protein sequence and consequently excluded from the dataset. The remaining superTranscripts assigned by both packages as non-coding were considered as candidate lncRNAs. Finally, to determine high-confidence lncRNAs in blue sharks, the clean reads of the candidate lncRNAs were mapped to the superTranscriptome with STAR v-2.7.10b [34], and gene expression levels were quantified (as transcripts per million, TPM) in Salmon v-1.10.1 [35]. Transcripts with TPM ≥ 1 were defined as high confidence lncRNAs. To identify putative orthologues of the predicted high-confidence lncRNAs in blue sharks, ncRNA sequences for five other shark species spanning three orders were downloaded from NCBI. These included another *Carcharhiniformes*, the small-spotted catshark (*Scyliorhinus canicula*) (RefSeq accession: GCF_902713615.1); one *Lamniformes*, the white shark (*Carcharodon carcharias*) (RefSeq accession: GCF_017639515.1); and three species of Orectolobiformes, the whitespotted bamboo shark (*Chiloscyllium plagiosum*) (RefSeq accession: GCF_017639515.1), whale shark (*Rhincodon typus*) (RefSeq accession: GCF_021869965.1), and zebra shark (*Stegostoma fasciatum*) (RefSeq accession (GCF_022316705.1). The predicted high confidence lncRNAs from blue sharks were aligned with those from these additional five species using the CRB-Blast package v-0.6.9-5 [36], and paired lncRNAs with a ≥60% identity and a cut-off value ≤ 1 × 10^−6^ were considered as putative orthologue lncRNAs. To identify lncRNAs that may act as putative miRNA precursors in sharks, the high-confidence lncRNAs identified in this study were aligned against known miRNA precursor sequences extracted from mirBase (Release 22.1, http://www.mirbase.org/, accessed on 24 March 2023) using BLASTn. Matching sequences with >90% identity and e-value < 1 × 10^−1^ were considered as plausible miRNA precursors.

### 2.3. Tissue-Specific lncRNAs

Tissue specificity of lncRNAs was determined using the preferential expression measure (PEM) index, calculated according to the TPM expression values by transcripts/tissues [37]. PEM is a single scalar metric that indicates the ubiquity or specificity of any given gene per tissue. PEM values range from 0 to 1, where genes that are tissue-specific have values of approximately 1. In this study, lncRNAs with PEM values ≥ 0.8 were considered TS, while those with TPMs greater than 0.5 and a TPM coefficient of variance less than 1 in all four tissues examined were identified as stably expressed lncRNAs.

### 2.4. LncRNA Differential Expression and Coding Target Genes

Clean reads from each tissue type were mapped to the assembled superTranscritptome with STAR v-2.7.10b [34], and raw read counts were obtained with Salmon v-1.10.1 [35]. The DESeq2 R package was used to identify lncRNAs with differential expression among the four different tissues [38]. LncRNAs with corrected *p* values (Benjamini–Hochberg false discovery, FDR) < 0.05 and an absolute value of log 2-fold change ≥1.5 were considered differentially expressed (DE). Given that numerous studies have reported a relevant role of lncRNAs in the regulation of protein-coding genes [39], we evaluated the potential interaction among mRNA targets and DE lncRNAs in blue sharks. There are two main approaches for predicting coding target genes of lncRNAs: cis or trans-target. Since a blue shark reference genome is not available, we selected the trans-target option. For this, the BLASTn algorithm was used to select protein-coding target genes complementary to DE lncRNAs (≤1 × 10^−6^ and identity ≥ 95%). The program LncTar [40] was used to confirm the relative stability of the interactions detected among lncRNAs and protein-coding genes, with a cut-off value of normalized free energy of −0.13.

### 2.5. RT-qPCR Validation

Four DE lncRNAs expressed in all the tissues were randomly chosen for RT-qPCR analysis (TRINITY_DN433_c0_g5, TRINITY_DN81282_c0_g1,TRINITY_DN4558_c1_g1, and TRINITY_DN8728_C1_G1). Reverse transcription was performed with a blend of random primers and oligo (dT) and iScript reverse transcriptase (iScript cDNA synthesis kit, Bio-Rad, Hercules, CA, USA) according to the manufacturer’s instructions. LncRNA-specific primers for each of the selected lncRNAs were designed with Primer3 Plus (https://www.primer3plus.com/index.html, accessed on 19 March 2024) (Supplementary Table S1). Quantitative real-time PCR was conducted using RT² SYBR Green ROX qPCR Mastermix (Qiagen, Germantown, MD, USA) and Rotor-Gene Q (Qiagen). qPCR cycling consisted of 95 °C for 4 min, followed by 40 cycles of 95 °C for 6 s and 60 °C for 45 s, with a final melting curve analysis at 95 °C for 12 s, 60 °C for 60 s, and 95 °C for 15 s. All RT-qPCR reactions were performed in triplicate, and beta-actin (GenBank: AY455921.1) and TRINITY_DN16185_c1_g2 genes were utilized as normalizers. TRINITY_DN16185_c1_g2 was selected from a subgroup of eight lncRNA genes that exhibited no differential expression in the RNA-Seq data among the four tissue types. The cycle threshold values from the qPCR expression data for these eight lncRNA genes and beta-actin across the four tissues were analyzed with NormFinder [41] and BestKeeper [42] to determine the most reliable reference gene for RT-qPCR. The fold changes in the expression of the selected lncRNAs in distinct tissues were estimated based on the 2^–ΔΔCt^ method [43]. The PCR efficiencies of each pair of primers were calculated with the LinRegPCR V. 2021.2 [44] package based on raw fluorescence data. Functional assessment of the identified DE lncRNAs was performed relative to the functional annotation of their protein-coding genes target. GO (Gene Ontology) biological process terms were determined with Cluster Profile v- 4.2.2 [45], and GO terms were considered as significant with a *p* adjusted-value (BH) < 0.05 and a fold enrichment > 2.

## 3. Results

### 3.1. De Novo Assembly Overview

Sixteen cDNA libraries from four tissues of blue sharks (Spleen 1–4, Liver 1–4, Muscle 1–4, and Kidney 1–4) were sequenced to obtain information of the identification and gene expression patterns of sharks lncRNA (NCBI BioProject accession PRJNA1139720). A total of 772,534,672 base pair (bp) pair-end raw reads were obtained, and after read trimming and exogenous decontamination, 755,635,033 clean reads remained, with a GC content and Q30 average for the samples of 46.51% (±0.77) and 93.73% (±0.72), respectively (Supplementary Table S2). A total of 218,543 assembled transcripts (encoded by 166,479 trinity “genes”) were obtained, with an average length of 731.97 bp (range of 201–35,984 bp), a N50 value of 1221 bp, and 45,534 ORFs predicted (≥100 aa). Assessment of the transcriptome completeness by BUSCO showed that nearly 99% of core genes were recognized: complete genes found (C), 98.7%; fragmented genes found (F); 0.9%; duplicated genes found (D), 22.4%; and missing genes (M), 0.4%.

### 3.2. Identification and Characterization of lncRNAs in Blue Sharks

**Figure 1:**
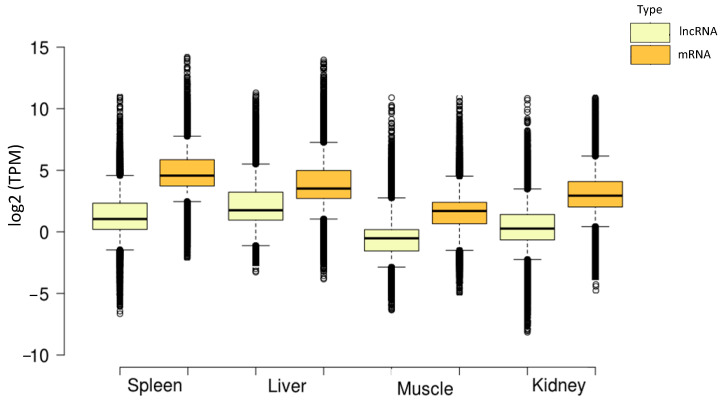
Gene expression (transcripts per million; TPM) distribution boxplots of long non-coding RNAs (lncRNA) and mRNAs per tissue type in the blue shark (*Prionace glauca*).

A total of 21,932 high confidence lncRNAs were identified in blue sharks, with a length ranging from 201 to 9311 bp (median and average lengths of 611 and 885 bp, respectively) (Supplementary Table S3). This number of lncRNAs is substantially higher than those previously reported for the whitespotted bamboo shark [23] and Australian ghostshark (*Callorhinchus milii*) [22], although the distributions of sequence lengths were comparable. The comparison with available ncRNA sequences of several shark species (i.e., white shark, whitespotted bamboo shark, whale shark, small-spotted catshark, and zebra shark) revealed that the majority of lncRNAs in blue shark (N = 21,920) are novel and have not previously been documented in any of these other shark species (Supplementary Table S4). The lengths of the lncRNAs sequences identified in blue sharks were shorter than the annotated protein-coding genes (median and average lengths of 754 and 987 bp, respectively), with a slightly higher GC content for protein-coding genes (46.34%) compared to lncRNAs (42.58%). Notably, lncRNAs in blue sharks demonstrated lower expression levels than protein-coding genes in all the tissues analyzed (Figure 1). Further, two lncRNAs in blue sharks were identified as potential precursors of two known miRNAs (TRINITY_DN10569_c4_g1: *Oryzias latipes*-mir-181b-1 and TRINITY_DN11922_c0_g4: *Homo sapiens*-miR-1244-4).

### 3.3. Identification of lncRNAs among Tissues

**Figure 2:**
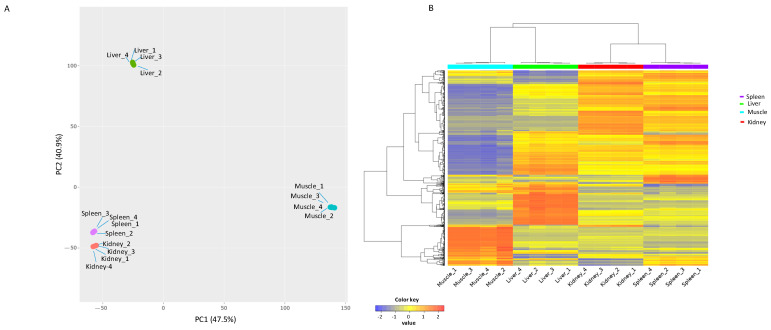
Principal component analysis (**A**) and heatmap (**B**) of high confidence long non-coding RNAs (lncRNAs) identified from blue shark (*Prionace glauca*) tissues.

To categorize the global expression patterns of the lncRNAs identified among tissues, a principal component analysis (PCA) (RNA-seq data normalized to relative log estimate) and heatmap (RNA seq data normalized to TPM) analyses were utilized. The four tissue types from blue sharks were distinctly clustered in the heatmap analysis in the first and second principal components (PC1 and PC2), explaining 88.4% of the variance (Figure 2).

**Figure 3:**
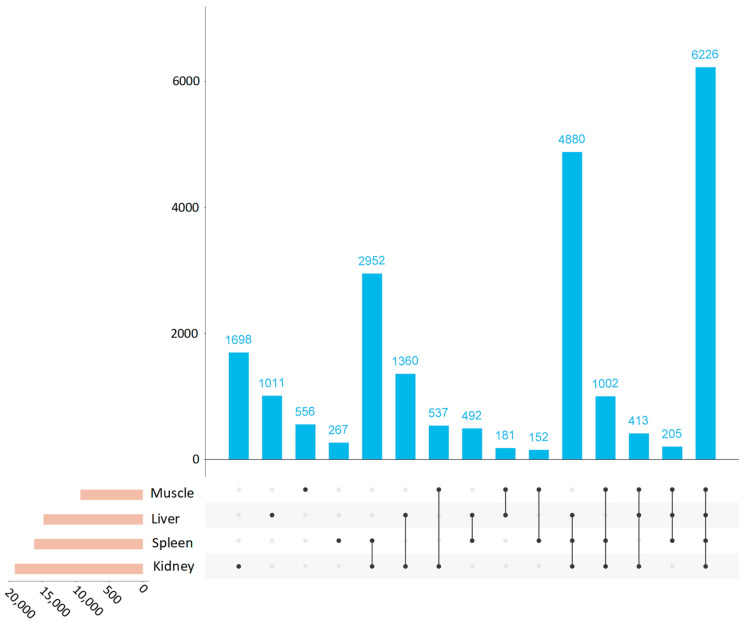
Upset plot showing the number of shared long non-coding RNAs (lncRNAs) expressed across blue shark (*Prionace glauca*) tissues.

**Figure 4:**
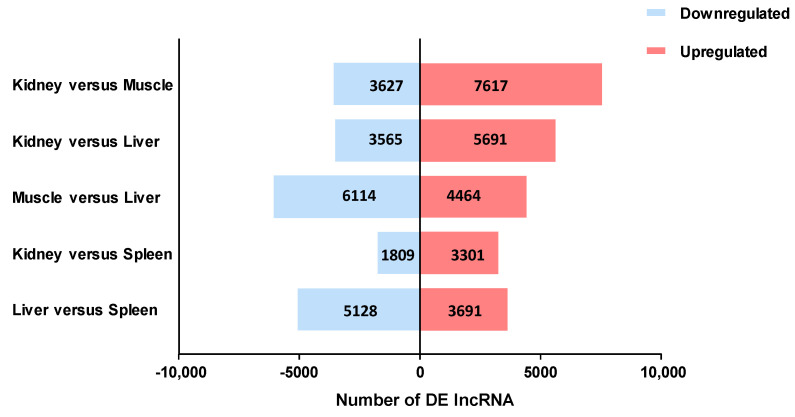
Total number of differentially expressed (DE) long non-coding RNA (lncRNA) in pairwise comparisons of blue shark (*Prionace glauca*) tissues.

A mean number of 14,821 lncRNAs were observed per tissue type, with only 6226 shared among all tissues (Figure 3). Most of the lncRNAs identified were expressed (TPM ≥1) in the liver (14,768), spleen (16,176), and kidney (19,068), while muscle showed the smallest number (9272). The kidney and spleen, key lymphoid organs in sharks, share a close pattern of lncRNA expression. A total of 45,007 DE lncRNAs were obtained between pairwise tissue type comparisons, with the greatest and least number of DEs found among kidney vs. muscle and kidney vs. spleen, respectively (Figure 4) (Supplementary Table S5).

**Figure 5:**
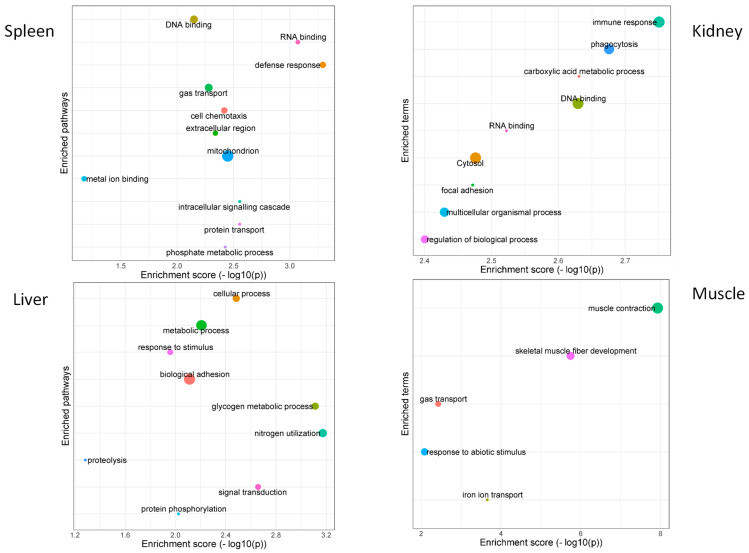
GO enrichment analysis of differentially expressed (DE) long non-coding RNA (lncRNA) protein target genes in blue shark (*Prionace glauca*) tissues. Graph size represents the number of GO gene associated.

An analysis of the protein-coding target genes of the total DE lncRNAs detected 454 protein-coding genes as trans-regulated and targeted by 1001 lncRNAs. GO enrichment analysis of these target genes across tissues showed diverse, relevant biological process terms, such as muscle contraction (GO:0006936) and skeletal muscle fiber development (GO:0048741) in muscle tissues; phagocytosis (GO:0006909) and immune response (GO:0006955) in kidney tissues; glycogen metabolic process (GO:0005977), signal transduction (GO:0007165), and nitrogen utilization (GO:0019740) in liver tissues and defense response (GO:0006952), RNA binding (GO:0003723), and defense response (GO:0006952) in spleen tissues (Figure 5).

### 3.4. Tissue-Specific lncRNA Expression

**Figure 6:**
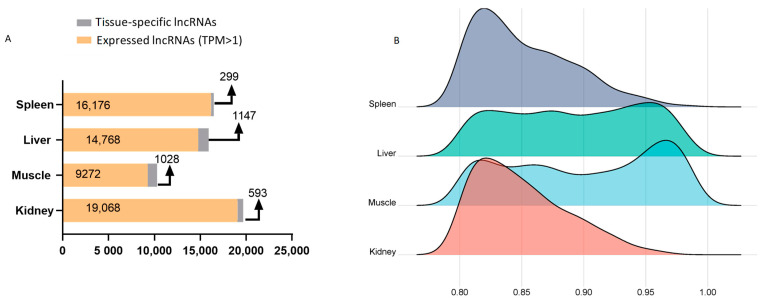
Number of tissue-specific (TS) and expressed lncRNAs across blue shark (*Prionace glauca*) tissues (**A**). Density plot of maximum-normalized preferential expression measure (PEM) values by tissue (**B**).

Using the PEM index, we found that liver (1147) and muscle (1028) tissues had the greatest numbers of TS lncRNAs, while kidney (593) and spleen (299) had the least. Further, a density plot of maximum-normalized PEM values shows similar patterns among the liver and muscle, as well as spleen and kidney tissues (Figure 6). In addition, a total of 8984 stably expressed lncRNAs were identified across all the tissues (Supplementary Table S6).

### 3.5. Experimental RT-qPCR Validation of lncRNAs

**Figure 7:**
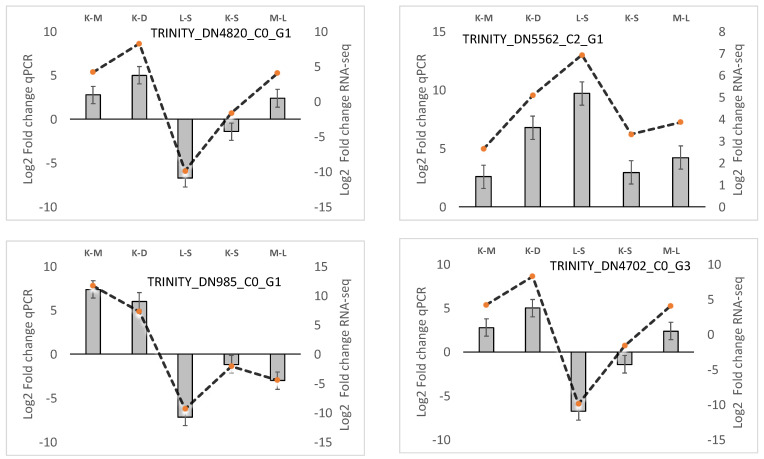
Validation of four lncRNAs in blue shark (*Prionace glauca*) using RT-qPCR. CT-qPCR raw data were analyzed using the 2^−ΔΔCt^ method using beta-actin and TRINITY_DN16185_c1_g2 as reference genes. Each column represents the mean of qPCR technical replicates ±SE, and broken lines represent RNA-seq fold change tissue values.

All of the primer pairs designed for the four DE lncRNAs chosen for qPCR validation had an efficiency between 94.57% and 98.02%, which are within the ideal range for qPCR analysis. The relative expression of the tissues DE lncRNAs was in agreement with RNA-seq results (Figure 7). 

## 4. Discussion

While a sizable amount of lncRNA research has been conducted on plant and animal model organisms [20,21], there is comparatively little information available on lncRNAs in non-model species, such as sharks. Numerous studies have shown that lncRNAs can modulate gene expression and participate in several physiological processes [39]. Studies on tissue-specific characterization and lncRNAs in sharks is very scarce compared to teleost fishes. Prior to this study, transcriptomics investigations on sharks focused more on identifying functional coding genes [11,12,13,14] rather than ncRNA genes, including lncRNA. In this research, we used ribosomal-RNA depleted RNA-seq on an Illumina HiSeq 2500 platform and a highly stringent data processing pipeline to discard transcripts with protein-coding potential, minimizing the inclusion of false positive lncRNAs in blue sharks. A total of 21,932 lncRNAs were identified across the kidney, spleen, liver, and muscle tissues of blue sharks. Compared to protein-coding genes, these lncRNAs were shorter with lower expression levels and GC content, which is concordant with reports for other vertebrate lncRNAs studies [46].

The lncRNAs identified in the blue shark in this study demonstrate a high degree of evolutionary novelty, with little conservation of lncRNA across shark species. Only 5% of the lncRNAs identified in blue sharks were also reported in other cartilaginous species (i.e., white shark, whitespotted bamboo shark, whale shark, small-spotted catshark, and zebra shark). Similarly, other studies have also shown low levels of sequence conservation of lncRNA across a wide taxonomic range [47]. Hara et al. [23] suggested a higher degree of lncRNA conservation within the cartilaginous fishes compared to teleost fishes. However, this hypothesis was based on a comparative blast approach including several teleost species with well-established genome annotation and only two shark species (brownbanded bamboo shark, *Chiloscyllium punctatum* and the cloudy catshark, *Scyliorhinus torazame*) with partial genome sequencing. Therefore, the greater conservation of lncRNAs in sharks in Hara et al. [23] may be due to an underestimation of the lncRNA diversity in the shark species (brownbanded bamboo shark and the cloudy catshark) involved in that study.

Comparative lncRNA expression analysis showed that functionally similar tissues (e.g., spleen and kidney) clustered closely together in terms of their global transcriptomic patterns. Both the spleen and kidney are recognized immune organs in teleost and cartilaginous fishes [48,49]. Since each organ has its own transcriptional pattern, several DE lncRNAs genes in functionally distinct tissues were expected. In fact, we found the largest number of differentially expressed lncRNAs gene among the kidney vs. muscle tissues. The main function of the muscles is movement, while that of the kidney is the regulation of physiological homeostasis and immune response. Furthermore, the regulatory efficacy of lncRNAs is dependent on their own expression. Numerous lncRNAs exhibited tissue-specific expression signatures, offering crucial hints about the unique roles that these molecules perform within cells/tissues. Moreover, there were stably expressed lncRNAs widely expressed in all tissue/cell types, establishing the fundamental transcriptome conservation for elementary biological functions. PEM index analysis revealed that the number of TS lncRNAs differs considerably among tissues and appears to be unrelated to the quantity of expressed lncRNAs in each type of tissue. The liver and muscle tissues showed the highest number of TS lncRNAs, perhaps driven by the need for more varied lncRNA repertoires or the existence of multiplex cell composition in these tissues. Conversely, the spleen and kidney tissues had the least number of TS lncRNAs, which could also be associated with the specialized functional roles of these tissues. The higher number of stably expressed lncRNAs (8984) compared to TS lncRNAs (3067) across all tissues might be indicative of the former participating in the regulatory response of a wide range of tissue functions and cell signals.

GO enrichment analysis of DE lncRNA protein-coding targets across tissues revealed that different biological process GO terms were enriched and associated with the specific biological functions of each tissue. Only two biological process GO terms (RNA and DNA binding) were shared among tissues (kidney and spleen). In the muscle, DE lncRNAs targeted protein-coding genes were involved in muscle system processes and gas and iron transport. Blue sharks have a long-distance dispersal across and between ocean basins [50]; therefore, lncRNAs are likely pivotal components in the modulation of muscle activities and muscle regeneration and differentiation necessary to undertake these migrations. In the kidney and spleen, the main GO terms involved responses to stress, cellular/metabolic processes, binding, and immune response. Both organs have been recognized as playing important roles in the immune and stress responses in sharks [51,52], which is consistent with the relevance and roles reported in several teleost species [53]. Additionally, the liver DE lncRNA protein-coding targets showed outstanding metabolic and homeostasis cellular process GO terms. The liver is a large, multi-lobed organ in sharks with key catabolic and anabolic functions [49]; therefore, lncRNAs could represent relevant contributors to the biological functions of the liver.

## 5. Conclusions

This research provides novel information on lncRNAs from multiple tissues in a shark for the first time, filling a gap in our knowledge and characterization of shark ncRNA. These data will provide a key resource for supporting future studies on the functional characterization of lncRNAs in sharks and serve as an important first step in understanding the adaptation of a sentinel marine species to environmental changes and stress.

## Data Availability

The RNA-seq from this study were deposited in the Sequence Read Archive (NCBI) No. PRJNA1139720. All data acquired and analyzed in this study are included in this paper and the Supplementary Information File.

## Supplementary Materials

The following supporting information can be downloaded at: https://www.mdpi.com/article/10.3390/life14091144/s1, Table S1: List of primers used in RT-qPCR. Table S2. Overview of all libraries sequenced. Table S3. High confident blue shark lncRNAs identified. Table S4. List of conserved Blue shark_lncRNAs and their Blast results. Table S5. List of DEGs between pairwise tissue types comparison. Table S6. List of stably lncRNAs across tissues.
